# From Midwife-Dominated to Midwifery-Led Antenatal Care: A Meta-Ethnography

**DOI:** 10.3390/ijerph17238946

**Published:** 2020-12-01

**Authors:** Bente Dahl, Kristiina Heinonen, Terese Elisabet Bondas

**Affiliations:** 1 Centre for Women’s, Family and Child Health, Faculty of Health and Social Sciences, University of South Eastern Norway, P.O. Box 235, N-3603 Kongsberg, Norway; 2 Metropolia University of Applied Sciences, Health Promotion, P.O. Box 4000, FI-00079 Metropolia, Helsinki, Finland; kristiina.heinonen@uef.fi; 3 Department of Nursing Science, University of Eastern Finland, Finland, Yliopistonranta 1, 70210 Kuopio, Finland; 4 Faculty of Health Sciences, University of Stavanger, P.O. Box 8600, N-4036 Stavanger, Norway; terese.e.bondas@uis.no

**Keywords:** antenatal care, midwife, meta-ethnography, meta-synthesis, prenatal care, qualitative research

## Abstract

Provision of antenatal care includes risk identification, prevention and management of pregnancy-related diseases, but also health education, health promotion, support and guidance to smooth the transition to parenthood. To ensure good perinatal health, high-quality, free and easily accessed antenatal care is essential. The aim of this study was to identify, integrate and synthesize knowledge of midwives’ experiences of providing antenatal care, attending to clients’ individual needs whilst facing multiple challenges. We conducted a meta-ethnography, which is a seven-step grounded, comparative and interpretative methodology for qualitative evidence synthesis. A lines-of-argument synthesis based on two metaphors was developed, based on refutational themes emerging from an analogous translation of findings in the included 14 papers. The model reflects midwives’ wished-for transition from a midwife-dominated caring model toward a midwifery-led model of antenatal care. Structural, societal and personal challenges seemingly influenced midwives’ provision of antenatal care. However, it emerged that midwives had the willingness to change rigid systems that maintain routine care. The midwifery-led model of care should be firmly based in midwifery science and evidence-based antenatal care that emphasize reflective practices and listening to each woman and her family. The change from traditional models of antenatal care towards increased use of digitalization no longer seems to be a choice, but a necessity given the ongoing 2020 pandemic.

## 1. Introduction

Good perinatal health has a major impact on public health, including the health of individuals, society and future generations [1]. To ensure good perinatal health, high-quality, free, and easily accessed antenatal care (ANC) is vital. According to the World Health Organization (WHO), antenatal care can be defined as *‘The care provided by skilled health-care professionals to pregnant women and adolescent girls in order to ensure the best health conditions for both mother and baby during pregnancy. The components of ANC include: risk identification; prevention and management of pregnancy-related or concurrent diseases; and health education and health promotion’* ([2], p. 1). ANC is also meant to provide support and guidance to parents and families to help ensure a positive experience for all. The organization of antenatal care is an important part of maternity care. It varies throughout the world but is generally provided within private and public health care in hospitals and health care centres [3]. Although an increasing number of women attend ANC appointments worldwide, global maternal health inequalities and the provision of services across and within countries still reflect low participation in some countries [4,5].

ANC dates back to the early 20th century, when it was developed as a means to prevent complications and educate mothers; over time, it included preparation for birth, based on principles from Dick-Read’s 1933 book, *Natural Childbirth* [6] and later from Lamaze’s 1955 book, *Painless Childbirth* [7]. In recent years, the inclusion of both parents in ANC has been recommended, reflecting a new focus on parenthood in addition to pregnancy and birth. ANC is usually provided to the woman and her partner on a routine basis, and is supplemented with parent education classes (PEC). The health care professionals working within ANC—mainly midwives, public health nurses and nurses, but also obstetricians and general practitioners—vary in terms of roles and responsibilities. Moreover, different ANC models are described, including traditional one-to-one individual care and various forms of group-based care [8].

For many women, initial or continued participation in ANC depends on whether they perceive it as a positive experience. Women expect that services will be of good quality and include continuity of care that is personalized, kind, caring, supportive and culturally sensitive; moreover, young families expect care to be flexible and respectful with regard to their need for privacy [3], and immigrant families have brought culture care challenges into the maternity care services [9]. Thus, while prevention of morbidity and mortality through effective ANC are key, the preservation of clients’ dignity and their continued participation is equally important [10].

Midwives tend to focus on caring for the pregnant woman, developing a relationship with her and preparing her for the birth of her baby [11]. Fathers and partners, on the other hand, often feel fearful and unsupported in the encounter with maternity care services [12]—with the corresponding risk that the father or partner is assigned the role of bystander or support person for the woman. There is increasing evidence of perinatal depression, starting early in pregnancy. A continuous trustful relationship with the midwife, identifying need for extended support may prevent long-term psychiatric illness [13].

To provide ANC according to women’s individual needs, health care providers must have sufficient resources and time to provide flexible and personalized appointments [3]. This, in turn, depends on organizational norms and values that recognize the importance of staff who respect women and approach pregnancy as a normal life event but are skilled at identifying and addressing complications. Models of care with an explicit epistemological status are important in order to implement evidence-based care [14]; however, Symon et al. [15] found that several ANC models lack an epistemological basis. Furthermore, the public sector has been squeezed by economic problems that have resulted in reduced preventive and promotive public health care, including ANC [16,17]. Thus, the introduction of new models, such as group-based care and digital training, seems to still be in a project phase, dependent on enthusiasts [18]. Moreover, the maternity care services, including ANC, have experienced an increased medicalized approach, including the use of routine guidelines and interventions. In this context, it may be challenging for midwives to care for pregnant women the way they wish. In their Australian study, Sidebotham et al. [16] found a general mood of disenchantment, disillusion and negativity among ANC midwives. Likewise, Catling et al. [17] describe a culture of powerlessness and midwives’ experiences of being constrained by their environment.

While antenatal care has largely remained unchanged, it is being challenged by the digital age and parents’ wishes, and the pandemic outbreak in 2020 has underlined the importance of implementing use of digital technology in ANC as soon as possible.

The digital age has invited both professional and private lay care initiatives, especially in birth and family preparation—and these include education that may not be evidence-based and create conflicts and contradictions. Thus, it is important to develop effective and meaningful evidence-based antenatal care that smooths the transition to parenthood while supporting the health of the woman and baby.

Reviews have explored women’s and health care providers’ experiences regarding uptake of routine antenatal services [3]; group versus conventional antenatal care [8]; midwife-led continuity models versus other models of care [19]; and alternative versus standard packages of antenatal care [20]. In this synthesis, we sought to explore how midwives experienced providing antenatal care, attending to their clients’ individual needs whilst facing multiple challenges: as such, the study design was aimed at identifying, integrating and synthesizing knowledge of midwives’ perceptions of providing antenatal care.

## 2. Materials and Methods

### 2.1. Design

Meta-ethnography is a grounded, comparative and interpretative methodology for qualitative evidence synthesis [21]. This approach was developed by sociologists Noblit and Hare [22], and has become an influential methodology in health and social care research. Meta-ethnography relies on what Geertz [23] referred to as ‘thick descriptions’, or detailed reporting of social or cultural events taking place in people’s lives; it also draws on Turner’s [24] theory of sociological understanding as ‘translation’. This methodological approach consists of seven overlapping and iterative phases (Table 1), and has the potential to synthesize qualitative studies: transcending individual studies, bringing new knowledge and thus facilitating new research questions and future interventions. The study was conducted according to the eMERGe meta-ethnography reporting guidance [25] (see Appendix A Table A1). The guidance developed to improve reporting quality in meta-ethnographies consist of 19 reporting criteria, covering all 7 phases in the meta-ethnographic method.

### 2.2. Inclusion Criteria and Search Strategy

We included empirical studies using qualitative methodologies focusing on midwives’ perceptions of providing ANC in normal pregnancy in high- and middle-income countries. Papers presenting different perspectives, e.g., pregnant women, general practitioners, obstetricians or nurses were included when it was possible to separate the perspectives. Papers using mixed methods were included if they presented qualitative data. The papers had to be written in English or Nordic languages and published in peer-reviewed scientific journals. We excluded quantitative studies, theoretical papers, reviews, master’s theses, dissertations and papers describing ANC in developing countries. Search filters limiting publication year were not used as we did not want to overlook relevant studies.

A literature search was conducted in April and May 2018, followed by an updated search in May 2020. Our primary literature search covered a multitude of databases, including: PsycInfo, PubMed, ProQuest, Wiley, Scopus, MEDLINE and CINAHL. The updated search was conducted in PsycInfo, ProQuest Social Science Premium Collection, Scopus, CINAHL and MEDLINE. Medical subject headings and text words used for the searches included ‘prenatal care’, ‘antenatal care’, ‘visit’, ‘midwife’ and ‘public health nurse’ combined with ‘qualitative’. Boolean operators AND, OR and NOT were used to combine the terms and truncations were used to ensure a sufficiently broad search. Furthermore, we searched the following journals to check that we did not miss articles that might have used other MESH terms: *Qualitative Health Research*, *Journal of Midwifery & Women’s Health*, *Journal of Obstetric, Gynecologic & Neonatal Nursing*, *Birth*, *Sexual and Reproductive Healthcare*, *BMC Pregnancy & Childbirth*, *Women & Birth, International Journal of Childbirth* and *Journal of Advanced Nursing*. In addition, we backtracked reference lists in the included articles. The responsibility for conducting the literature searches was divided between the authors, and experienced librarians assisted in finding relevant key terms and performing the searches.

### 2.3. Search Outcome

**Figure 1:**
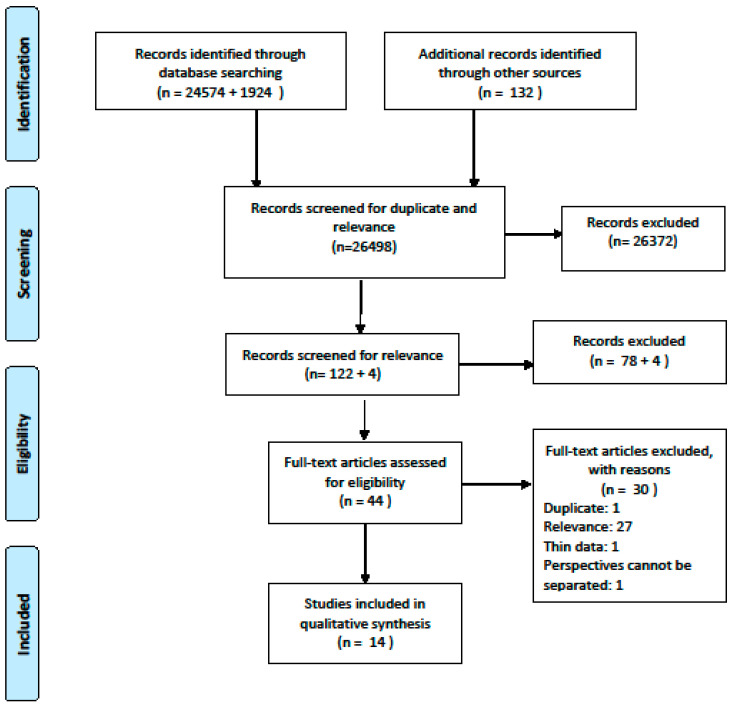
PRISMA Flow Diagram.

The literature search is described in detail in the Preferred Reporting Items for Systematic-Reviews and Meta-Analyses (PRISMA) flow diagram [26] (Figure 1). We began the screening process by sorting out duplicates and irrelevant publications based on article titles. Abstracts were then reviewed, and irrelevant publications—e.g., articles focusing on ANC for vulnerable groups and papers employing unsuitable methodologies—were excluded. The remaining 44 papers were read in full, and were screened according to the inclusion and exclusion criteria. This resulted in the exclusion of another 30 papers. The updated literature search resulted in four articles to be screened, but none were found relevant. The screening process was conducted by all authors, and commenced with individual readings that were then followed by team discussions and achievement of consensus. The updated search was screened by the first author.

### 2.4. Quality Appraisal

All authors were involved in assessing the included papers, guided by a checklist from the Critical Appraisal Skills Programme (CASP) [27]. This checklist is designed as a pedagogical tool, and does not suggest a scoring system. For our research team, it worked as a systematic reminder of issues related to study quality, including study aim, methodology, recruitment strategy, data collection, reflexivity, ethical issues, data analysis, statement of findings and study value. One author assessed all articles that were eligible for inclusion, and the other authors shared the articles between them to ensure that two authors assessed all articles independently. We arranged a Skype meeting to discuss minor disagreements and reach a final consensus on inclusion. We considered all studies to have sufficient quality to be included in our synthesis, and provide a detailed list of the quality appraisal in Table 2.

### 2.5. Data Extraction and Synthesis

We started the analysis process with deciding the focus of the synthesis and continued with a thorough literature search, identifying and selecting relevant studies. All researchers were involved in this process. The next phase involved a more intense and repeated reading of the studies. We developed a table that described the study aim, sample characteristics, research design, data collection, data analysis and key findings in order to provide context (Table 3).

We continued by immersing ourselves in the data and noting the interpretative metaphors and/or core concepts throughout the studies. This process was performed by the researchers individually and in pairs, and was facilitated by the use of a Microsoft Word table to list and compare the first- and second-order concepts across the studies. We incorporated the findings from the studies into one another by analogue translation to form new third-order refutational concepts. The analysis process was iterative and involved moving back and forth in the data, comparing and contrasting the findings from the individual studies and translating them into one another. Finally, the synthesis of translation allowed themes to emerge and an overarching lines-of-argument model based on two metaphors was created. We used the GRADE CERQual [28] (Table A2) to assess the confidence in evidence of the study findings. The tool provides a method for assessing the confidence of evidence from reviews of qualitative research and consists of four components: methodological limitations, relevance, coherence and adequacy of data. The CERQual assessment demonstrated moderate confidence in the themes. It is therefore likely that the themes are reasonable presentations of midwives’ experiences of providing ANC.

## 3. Results

It is important to consider the varying settings of review studies [29]. In this meta-ethnography, the included studies concerned antenatal care in four countries, across different types of public or private antenatal care services, over a period of more than 20 years. We included three studies from Sweden with midwife participants from urban and rural areas, practising in small and large primary health care and hospital clinics. The United Kingdom contributed five studies from various parts of the country, including different types of antenatal care facilities. From Australia, five studies were included, from different clinics in different parts of the country. Finally, though only one study was included from Canada, it included several maternity clinics, hospital pre-birth registration clinics and public health programmes. Altogether, the papers included 328 midwives or nurse-midwives, with sample sizes varying between five and 56 participants. Most papers used thematic analysis, including some form of interview as a data collection method. The studies described midwives’ experiences related to offering ANC and developing a relationship with the women in their care. They also described the importance of engaging fathers, providing parenthood education, caring for minority ethnic women, communication and the use of information and communications technology (ICT).

**Figure 2:**
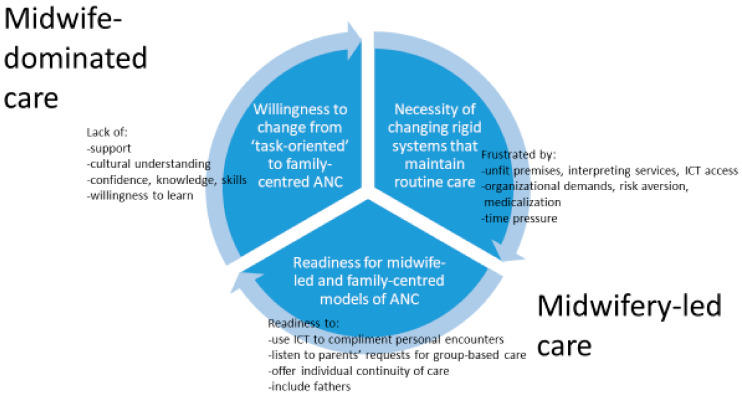
Antenatal care, from a midwife-dominated toward a midwifery-led perspective.

We developed a model based on two metaphors that captured our overall findings: of midwives practising from a *midwife-dominated model of care* but shifting toward a *midwifery-led model of care*. This model developed out of the refutational findings that midwives need to change the rigid systems that maintain routine care; their attitude towards changing from routine ANC provision to a family-centred caring approach; and their readiness to implement new caring models (Figure 2). The midwife-dominated model of care was characterized by routine procedures and rigid systems; in this context, supporting the pregnant woman and her partner in their journey toward parenthood was not a priority. However, it emerged that the midwives wanted to provide more personalized, evidence-based and family-centred care, including flexible models of ANC, making use of digitalization and listening to the woman and her partner: representing a shift toward a midwifery-led model of care.

Our theoretical model cannot serve as a model for care but it may elucidate some aspects regarding the ‘what, why and how’ of caring models used to provide high-quality ANC for women and their families. Thus, it illustrates and provides an increased understanding of the phenomenon, contributing to the body of knowledge in midwifery science. In order to change from midwife-dominated to midwifery-led care, it is vital to explore obstacles influencing caring practices, challenge factors maintaining routine care and encourage new ways to improve practice. Midwifery theories can provide the profession with a lens that helps define the core elements and aims of midwifery practice: this is an important step toward developing a ‘midwifery language’ and a model describing midwifery-led care.

### 3.1. Willingness to Change from Routine ANC to Family-Centred ANC

The participants described a willingness to change from a routine ANC approach toward providing family-centred ANC. However, they described that this change was challenged by a lack of support from their colleagues, their own lack of cultural understanding and language barriers, and their own lack of confidence, knowledge and skills to support new practices. Finally, they noted the parents’ resistance to changes in ANC.

#### 3.1.1. Lack of Support from Colleagues

Some midwives considered changing their current ANC approach, from providing traditional, one-to-one care to providing a form of group-based ANC [30]. However, this kind of shift in practice would require increased collaboration with other professions [31], and all of the staff would need to be engaged in the implementation and provision of this new form of ANC. The midwives noted a lack of support from colleagues or managers concerning introducing group-based care: *‘There was a midwife who wanted to try it out at our clinic, but no other colleague did; therefore, they did not help her book patients to her group’* [30].

#### 3.1.2. Lack of Cultural Understanding

Midwives found it difficult to balance professional conduct and cultural traditions when encountering immigrant or minority ethnic women [32,33]. Divergent expectations regarding ANC created tension, and cultural and religious practices sometimes clashed with routine care [32]. Some found it difficult to support women’s cultural and religious practices within the NHS maternity care system [32,33]. They were concerned about antenatal practices in Muslim culture, and found family members attending the consultations to be barriers to developing the midwife–woman relationship: *‘Because you never get to know them like you do, other ladies. You know… they’re keeping them back, I think, a little bit really’* [33]. Language barriers caused difficulties in communication, establishing relationships and providing care for minority ethnic women [30,31,32,33,34]. In situations where midwives were unable to communicate with their clients without having an interpreter present, communication became fragmented; furthermore, it was difficult to provide personal care while interpreters were physically present during medical examinations.

#### 3.1.3. Lack of Confidence, Knowledge and Skills to Support New Practices

Midwives and other staff considered it important to offer services in a culturally sensitive manner [35]. However, studies reported a lack of knowledge and confidence related to the introduction of group-based antenatal care and parenthood education models [30,31]. To support a group-based process, midwives needed a new set of pedagogical skills [31], and this sometimes resulted in a reluctance to try out new models. However, some midwives described how, after parents requested the use of new models, they became curious and were willing to give them a try. The use of ICT was another topic creating frustration. Although the midwives were familiar with the use of ICT to document medical information, they were more reluctant to use it to communicate with clients, arguing that they needed knowledge, training, social support and guidance to confidently use ICT for such purposes [18]. They feared that these platforms could be misused and difficult to control, and, according to one midwife, such technologies would provide *‘an avenue for raving nutters’* [18]. Consequently, they lacked motivation to use them in regular ANC and were worried that the use of ICT would endanger their ability to assess their clients’ condition, leading to poor care. Some midwives perceived social media as ’dangerous’, arguing that it had no place whatsoever in health care. However, younger midwives were less reluctant to use social media professionally than older midwives, suggesting that age may have influenced this response [18].

#### 3.1.4. Lack of Willingness to Learn about and Try Group-Based Care

The use of group-based antenatal care was described as timesaving, as it allowed health care providers to give information to several parents simultaneously, reducing their workload. However, some midwives expressed resistance toward group-based care, as it demanded engagement and time: *‘I do not want to involve myself in something new right now’* [30]. Others argued that it was difficult to talk about sensitive matters in a group and were worried that they would be unable to identify social problems in this setting; they claimed that some parents were uncomfortable with or lacked interest in group-based care. Furthermore, they said that group-based care was particularly unsuitable for immigrant women, arguing that there is no tradition in their culture with groups [30].

### 3.2. Necessity of Changing Rigid Systems that Maintain Routine Care

The participants described rigid systems that maintained routine care, such as unfit premises, ICT and interpreting service problems. Furthermore, organizational demands, risk aversion, medicalization and unsuitable recording systems obstructed their ability to provide personalized care. Time pressure and cultural misunderstandings were also described; however, in these instances, midwives still tried to prioritize the woman and her partner.

#### 3.2.1. Frustrated by Unfit Premises, Restricted Access to ICT and Interpreting Service

The opportunity to provide maternity care in homely and comfortable locations [36], convenient to women’s homes or workplaces and close to bus routes or free parking, was important to the midwives. They also felt it was essential that the location was welcoming and did not convey an impression of medicalization: *‘Women become pregnant, and you shouldn’t make a pathology out of it’* [35]. Small and unfit premises were considered obstacles to clients’ need for privacy [35] and prevented midwives from providing group-based care [30]. Standardized appointment lengths also impaired the situations and inhibited the midwives from conducting personalized care [32]. One study [18] found that telehealth was not an option in ANC and that clients were not allowed to access their electronic care records—they were, however, given physical copies of their records. Studies also described how language barriers resulted in fragmented communication and required use of interpreters [32,34]. Appointment lengths were usually standardized and did not allow for flexibility—*‘they might not be long enough or flexible to get all that information in’* [32]—and accessing professional interpreting services after hours was complicated. This created a logistical barrier to providing personalized care [32].

#### 3.2.2. Frustrated by Rigid Structures, Risk Aversion and Medicalization

The importance of developing high-quality and cost-effective programmes in terms of cost-cutting efforts was described [31]. One study depicted midwives as risk-negotiators, stressing how midwives find it important to focus on normality and resist systemic restraints, such as guidelines that are often not evidence-based [37]. Adhering to the structure of the pregnancy EDC wheel to provide routine task-oriented ANC became symbolic of midwifery-centred care rather than woman-centred care: *‘States the gestational weeks “you’re 28 weeks today” … all questions directed to the gestation and the tests appropriate for this time as per the protocol’* [38]. Furthermore, screening was considered routine, women were given little information and consent was seldom requested [39].

#### 3.2.3. Frustrated by Time Pressure and Cultural Misunderstandings

Limited time and poor staffing challenged midwives’ ability to deliver equitable services to all women. Time-saving efforts included offering ANC consultations with standardized appointment lengths and using computers to record information during appointments [32]. However, the midwives explained that time-poor actions—like writing while the women were talking or using computers to record information during appointments—negatively influenced communication. *‘You have to be willing to ask that question too, to leave an opening for a conversation about what things a woman might be worried about’* [40]. Limited time due to many appointments each day also influenced the ‘tone’ in the interviews [39] and restricted midwives regarding discussing options and completing birth plans [41]. This lack of time was therefore associated with routine antenatal care [38]. Busy clinics also hampered midwives’ opportunities to discuss options with women [41], and the use of standardized appointment lengths limited their ability to provide personal care [32]. Finally, midwives working with minority ethnic women found it difficult to provide holistic and woman-centred care since their clients sometimes rejected this approach, preferring their midwives to have a more administrative or task-oriented role [33]. Nevertheless, midwives felt that programmes should correspond to the assignment and demands of the participants [31]; they therefore employed strategies to counteract time pressure, such as trying not to look too busy in front of the women [40].

### 3.3. Midwives’ Readiness for Midwifery-Led and Family-Centred Models of ANC

The participants described a burgeoning desire to transition toward midwifery-led and family-centred models of ANC. The midwives described a readiness to use ICT as a complement to personal encounters, and saw the importance of listening to parents’ wishes for group-based care and personalized individual encounters, as well as including the fathers and family members in a more productive way.

#### 3.3.1. Readiness to Use ICT to Complement Personal Encounters

The midwives were aware of their clients’ use of mobile phones and apps and their information-seeking on the internet, and argued that even their lowest-income clients have access to the internet [18]. They said that they often had to rush through the information in the consultations and were willing to use ICT to complement this information provision. In situations where they must communicate remotely with their clients, they said they would consider using Skype, as this medium enabled face-to-face communication. They also found technology useful for engaging fathers, and said they would be more likely to direct them to useful websites to access information about pregnancy and birth: *‘They quite like using technology so I’m more likely to give them a slideshow or websites to look at…’* [34].

#### 3.3.2. Readiness to Listen to Parents’ Questions about and Requests for Group-Based Care

Some midwives said they were aware of and open to the views and choices of parents [42]. Others supported the view that group-based care provided an opportunity for parents to become friends, support each other and establish networks between mothers-to-be [30,31]. Moreover, group-based parenthood education was deemed important with regard to meeting the needs of today’s parents-to-be: *‘If the health and welfare authorities cannot provide them with what they need, they will look for it elsewhere…’* [31]. When conducting group-based ANC, midwives were encouraged to listen to parents’ questions and use them as a starting point [30]. Thus, group-based ANC was a good alternative for parents who already had considerable knowledge but needed help to sort out the body of information. Group-based approaches also allowed parents to develop discussions and create relationships with other parents in the same situation [30].

#### 3.3.3. Readiness for Individual Continuity of Care

The opportunity to provide emotional support was important for midwives, as it enabled them to develop a meaningful relationship with the pregnant woman [30,35,37], situate their care within the woman’s life context and position her as an active partner in her care rather than a passive recipient [35]. Some midwives considered involving women as active partners to be an essential feature of high-quality care: *‘I think if a person does feel very involved in their own prenatal care… that does encourage their motivation to be as healthy as possible’* [35]. Others argued that individual meetings were more important than group meetings [30] and that they preferred to see women in person to assess their condition [40]. By contrast, some midwives conveyed a lack of interest [42], seemingly acting as representatives of the system performing a routine job [39]. Moreover, the agenda of the ANC visits was sometimes based on midwives’ needs rather than the women’s needs, resulting in structured conversations and midwives dominating the encounters [38,42].

#### 3.3.4. Readiness to Include Fathers and Family Members

Midwives found it important to involve partners in ANC consultations [31,34]; according to some, continuity of care was critical in this regard [34,36,40]. To include fathers, it was important to offer flexibility with scheduling of appointments [34] and provide culturally sensitive ANC [35]. This was sometimes difficult, due to lack of knowledge and differing beliefs about fathers’ parenting roles [33,34] and time and funding constraints [34]. Some also found that language barriers and family members attending consultations contributed to challenges when working with fathers from non-Western cultures [33,34]. Many midwives said they had not received any formal training in working with fathers and requested father-focused education to improve their care provision. *‘I think, that it should become part of our education, how to integrate both parents when it comes to learning about pregnancy and delivery and childcare, so people feel a lot more confident’* [34]. They argued that targeted education would enable them to include more father-specific information, and they believed that the use of technology was a good strategy with which to engage men.

## 4. Discussion

Based on our analysis of 14 primary articles, we used the midwives’ perspectives to develop the lines-of-argument synthesis of a transition from a midwife-dominated toward a midwifery-led model of care. This synthesis reflects challenges at structural, societal and personal levels related to providing antenatal care, but also a growing interest in listening to parents’ needs and a readiness to implement new, flexible and individualized caring models, including a cautious interest in implementing digital tools.

Our findings show that midwives believed ANC services should be welcoming, locally provided, well-organized and cost-free [30,35,36]. However, in their clinical practice, they struggled to provide ANC within rigid structures focused on time- and cost-saving, medicalization and risk aversion [31,32,35,36,37,39]. Consequently, rather than providing individualized and midwifery-led care in the one-to-one-encounter—in accordance with their ideas of best practice—they ended up providing midwife-dominated care. Instead of listening to women and their partners, they offered standardized information, guidance and task-oriented care. In these situations, it was difficult to prioritize informed decision-making about care and treatment in partnership with the women due to heavy workloads and a lack of time.

According to family-centred maternity care (FCMC), ‘family’ is defined as those persons providing familial support [43]. Most midwives said they included the woman’s partner or family in their ANC provision; nevertheless, some appeared to focus primarily on the midwife–woman relationship in the individual encounter [30,35,37]. Similar findings are described elsewhere [44], underlining that there is a need for updated models of ANC that include partners and family. A central goal of FCMC is to strengthen the confidence of new parents, and supporting and encouraging new parents throughout pregnancy and the postpartum period builds trust in their own abilities [45]. For midwives, this entails finding ways to include partners and family. Some midwives believed the use of ICT would make it easier to include fathers but were reluctant to use ICT and social media themselves [18,34]. The present 2020 pandemic is likely to change traditional caring models, and an increase in digitalization is expected to be permanent. Thus, midwives providing ANC will have to find new ways to work. One study [46] showed that connected care visits supplemented by telephone or online portal visits, in which clients were given the opportunity to voice their concerns, resulted in increased client satisfaction. Today, parents are used to accessing information on the internet, and for fathers, the opportunity to connect with Facebook friends during the transition to parenthood has been associated with increased parental adjustment [47]. Similarly, the use of apps and social media platforms has the potential to influence pregnancy, motherhood and parenting practice, and thus traditional maternity care models should accommodate electronic devices [48]. Increased use of ICT in ANC can improve information sharing, and midwives can refer their clients to websites offering evidence-based information. Furthermore, when parents perform tasks they can do on their own, their sense of competence and autonomy is likely to improve.

One interesting finding concerned midwives’ attitudes about the implementation of new models for ANC. Many midwives argued that high-quality ANC requires individual encounters with women in order to develop a midwife–woman relationship [30,35,37]. They feared that the introduction of group-based models would jeopardize this relationship, and presented a variety of reasons against implementing group-based care. Digital consultations, e.g., Skype or Zoom may be used to provide individual and group-based ANC, but should be succeeded by research reviewing the literature that explores the implementation of such models and where necessary undertaking research that could add to the literature. The uncertainty and unwillingness demonstrated in our findings concerning the implementation of group-based care may demonstrate a lack of knowledge and genuine uncertainty about new models. It may also demonstrate a taken-for-granted understanding of best practice, representing values embedded in work culture and daily practice [49]. In a midwife-dominated approach to providing routine ANC, midwives may fail to see that the care they offer has a paternalistic overtone—i.e., that they know what is best for the woman and her family. Encounters with minority ethnic women and their families were sometimes described as challenging, and midwives found it difficult to accept and adapt cultural traditions and religious practices within the NHS healthcare system. According to core principles of FCMC [43], as well as the NICE guidelines for uncomplicated pregnancies [50], patient autonomy, respect, dignity and kindness are important factors when caring for pregnant women and their families. Both guidelines underline that informed decisions about care and treatment should be made in partnership between women, their families and their health care providers. Furthermore, in their core document ‘Philosophy and Model of Midwifery Care’, the International Confederation of Midwives (ICM) [51] states that midwives should respect ethnic and cultural diversity and provide care based on ethical principles, including the principle of respect for human dignity. However, to provide high quality, personalised and culturally sensitive care midwives must acknowledge cultural differences and traditions. Peters et al. [10] developed a model of midwifery practice underlining the importance of developing a relationship with women. Our findings demonstrate that this relationship is an important but challenging factor for midwives when providing ANC for minority ethnic women. Studies have demonstrated that the midwife-woman relationship is most successful when midwives and women share fundamental life philosophies and values [52]. The encounter with ethnic minority women may challenge midwives’ personal values and beliefs, creating feelings of uncertainty and ambivalence. However, midwives are provided with an opportunity to turn to their ‘blind spots’ in the encounter with women whose traditions, values and requests for care are unfamiliar. Thus, challenging encounters may provoke moral reflection and a possibility to develop culturally sensitive care. Thus, we argue that there is a need to develop sound, epistemologically based models for ANC in order to provide high-quality care. Studies have shown that it is difficult for midwives to promote normal births in a medicalized environment [53]; it is therefore unsurprising that midwives find it difficult to maintain a health-promoting focus on pregnancy in a working environment that is also focused on risk-assessment.

According to Leap [54], designing health care provision for groups instead of individuals is a relatively new idea. Traditionally, group activities for women during their childbearing years have predominantly been offered through antenatal education programmes or new mothers’ groups. More recently, the importance of antenatal groups that promote social support and the sharing of information have been highlighted [54]. It is vital to examine whether group care contributes to women’s activation and empowerment, and whether women receiving this style of care have access to the same level of information from care providers as those receiving standard one-to-one care [8]. The authors of the study conclude that future research should be conducted to find the best model for group antenatal care and to explore whether partners should be encouraged to attend or if women-only groups are more beneficial.

### Strengths and Limitations

Empirical variation within the sample is important in order to determine a study’s transferability [55]. We included two studies published in 1996 and 1998. These studies provided historic elements regarding differences and similarities in midwives’ perceptions of providing antenatal care. The remaining studies were published between 2006 and 2018. Altogether, our sample included 328 midwives from 4 different countries and we determined that our sample provided sufficient data to meet the study’s objective. The decision to omit studies published in low-income countries was deliberate, and we are aware that this influenced our findings.

We conducted a comprehensive search strategy in 2018 and updated the search in 2020. However, we are aware that our sample may not be complete. Our keywords may not have been comprehensive and inclusion criteria may have resulted in the loss of relevant information. Nevertheless, we found that the use of eMERGe meta-ethnography reporting guidance [25] and the CERQual Qualitative Evidence Profile [28] improved the study’s transparency and trustworthiness. Furthermore, our research group consisted of three persons with different international health care backgrounds (two public health nurses and one midwife) and experience. This provided a solid knowledge base and a varied perspective on antenatal care.

## 5. Conclusions

We developed the lines-of-argument synthesis of two metaphors of a *midwife-dominated model of care* shifting toward a *midwifery-led model of care* based on the refutational findings of midwives’ needs to change rigid systems that maintain routine care, their willingness to change from a routine ANC to a family-centred caring approach and their readiness to implement new caring models. The findings described midwives’ experiences of providing ANC and developing a midwife-woman relationship. They also described the importance of engaging fathers, providing culturally sensitive care and including digital tools in ANC. However, to move from midwife-dominated caring models (characterized by routine and task-oriented care) to midwifery-led caring models (characterized by shared decision-making and reflective practices, listening to each woman and her partner and family), practice should be firmly based in evidence-based knowledge.

## Figures and Tables

**Table 1:** Steps in meta-ethnography.

1	Getting started—identifying the topic of the study and defining the aim
2	Deciding what is relevant to the initial interest—including relevant studies, describing search strategy and criteria for inclusion and exclusion
3	Reading the studies—noting studies’ interpretative metaphors through repeated readings
4	Determining how the studies are related—determining the relationship between the studies by creating a list of key metaphors (themes, concepts, phrases, ideas) and assessing whether the relationships are reciprocal (i.e., findings across studies are comparable), refutational (findings stand in opposition to each other) or represent a line of argument
5	Translating the studies into one another—comparing metaphors and their interactions within single studies and across studies, and at the same time protecting uniqueness and holism
6	Synthesizing translations—creating a new whole from the sum of the parts, enabling a second-level analysis
7	Expressing the synthesis—finding the appropriate form for the synthesis to be effectively communicated to the audience

**Table 2:** Quality assessment of included studies.

Study	1	2	3	4	5	6	7	8	9	10
Alden et al., (2008)	YY	YY	YY	YY	YY	Y-	YY	YY	YY	YY
Andersson et al., (2014)	YY	YY	YY	YY	YY	Y-	YY	YY	YY	YY
Aquino et al., (2015)	YY	YY	YY	YY	YY	Y-	YY	Y-	YY	YY
Browne et al., (2014)	YY	YY	YY	YY	YY	--	YY	YY	YY	YY
Dalton et al., (2014)	YY	YY	YY	YY	YY	--	Y-	Y-	Y-	YY
Dove et al., (2014)	YY	YY	YY	YY	YY	Y-	YY	YY	YY	YY
Goodwin et al., (2018)	YY	YY	YY	YY	YY	--	YY	YY	YY	YY
McCourt (2006)	YY	YY	YY	YY	YY	--	--	YY	YY	YY
Olsson et al., (1996)	YY	YY	YY	YY	YY	Y-	Y-	YY	YY	YY
Proctor (1998)	YY	YY	YY	YY	YY	--	-Y	YY	YY	YY
Rominov et al., (2017)	YY	YY	YY	YY	YY	YY	YY	YY	YY	YY
Sword et al., (2012)	YY	YY	YY	YY	YY	YY	YY	YY	YY	YY
Whitford et al., (2014)	YY	YY	YY	YY	YY	--	YY	YY	YY	YY
Wright et al., (2018)	YY	YY	YY	YY	YY	-Y	YY	-Y	YY	YY

Abbreviations: Y = Yes; N = No; - = Cannot tell. Assessment questions: (1) Was there a clear statement of the aims of the research? (2) Is a qualitative methodology appropriate? (3) Was the research design appropriate to address the aims of the research? (4) Was the recruitment strategy appropriate to the aims of the research? (5) Was the data collected in a way that addressed the research issue? (6) Has the relationship between researcher and participants been adequately considered? (7) Have ethical issues been taken into consideration? (8) Was the data analysis sufficiently rigorous? (9) Is there a clear statement of findings? (10) How valuable is the research?

**Table 3:** Characteristics of included studies.

Author (Year) Country	Aim	Sample & Setting	Methodology	Methods for Data Collection & Analysis	Key Findings
Ahlden et al., (2008) Sweden	To describe perceptions of parenthood education among midwives and obstetricians in charge of antenatal care.	Midwives (*n* = 13) Obstetricians (*n* = 12) Large and small ANC clinics: Rural and urban areas, geographical distribution in Sweden, and annual ANC meeting	Qualitative phenomeno graphic approach	Focus group interviews	Support in transition to parenthood was important. Parenthood education should focus on awareness of the expected child, confidence in the biological processes and the change of roles.
Andersson et al., (2014) Sweden	To investigate and describe antenatal midwives’ perceptions and experiences of their current work, focusing on their opinions about group-based antenatal care.	Midwives (*n* = 56) Antenatal clinics all over Sweden	Mixed methods	Interviews with closed questions and comments Descriptive statistics and content analysis	Midwives were satisfied with their work, but had reservations concerning time constraints and parental classes. Many expressed interest in group-based care, but expressed personal and organizational obstacles to implementing the model.
Aquino et al., (2015) UK	To explore midwives’ experiences of providing care for Black and minority ethnic women.	Midwives (*n* = 20) NHS Antenatal clinics in North West England	Qualitative design	Semi-structured interviews Thematic analysis	Midwives found it difficult to communicate with women whose English was limited. They described a mismatch between midwives’ and women’s expectations of care and highlighted the necessity of inter-agency collaboration to address holistic care.
Browne et al., (2014) Australia	To explore midwives’ communication techniques intended to promote a wellness focus in the antenatal period.	Midwives (*n* = 14) Hospital/community settings in two states, including high-risk	Qualitative design	Focus group interviews	Midwives used health-promoting strategies in their work as an effort to reduce women’s anxiety and promote wellness-focused care.
Dalton et al., (2014) Australia	To investigate midwives’ attitudes toward and experiences of ICT use to identify potential causal factors that encourage or inhibit their usage in ANC.	Midwives (*n* = 19) Antenatal education in a metropolitan hospital		Semi-structured interviews, focus group interviews and survey Thematic and statistical analyses	Midwives recognized potential benefits of ICT use to deliver pregnancy-related health information, but had reservations about their use in everyday work.
Dove & Muir-Cochrane (2014) Australia	To examine how midwives and women within a continuity of care midwifery programme conceptualized childbirth risk and the influences of these conceptuali zations on women’s choices and midwives’ practice.	Midwives (*n* = 8) Obstetrician (*n* = 1) Women (*n* = 17) Community-based continuity of midwifery care programme	Critical ethnography	Semi-structured interviews and observation	Midwives assumed a risk-negotiator role in order to mediate relationships between women and hospital-based maternity staff. This role relied on the trust engendered by their relationship with the women.
Goodwin et al., (2018) UK	To explore the relationship between first-generation migrant women and midwives focusing on factors contributing to these relationships and their effect on the caring experience.	Midwives (*n* = 11) Pakistani women (*n* = 9) Community-based antenatal clinics	Focused ethnography	Semi-structured interviews Observation Thematic data analysis	The midwife–woman relationship was important for participants’ experiences of care. Social and ecological factors influenced the relationship, and marked differences were identified between midwives and women in their perceived importance of these factors.
McCourt (2006) UK	To explore the nature of information giving, choice and communication with pregnant women, in both conventional and caseload midwifery care.	Booking visits with pregnant women (*n* = 40) and midwives (*n* = 40) Hospital clinic GP clinic Women’s homes	Qualitative design	Non- participant observation Structured and qualitative analysis	Interactional patterns differed according to model and setting of care. A continuum of styles of communication were identified, and were more formal in conventional care than in caseload midwifery care.
Olsson et al., (1996) Sweden	To describe antenatal ‘booking’ interviews regarding content and to illuminate the meaning of the ways midwives and expectant parents relate to each other.	Booking visits with midwives (*n* = 5), women (*n* = 5) and fathers (*n* = 2) Midwifery clinics at five urban and rural primary care health centres	Qualitative design	Video-recorded booking interviews Content analysis and phenomeno logical hermeneutic analysis	Two perspectives of antenatal midwifery care, obstetric and parental, operated alternately and in competition within the interviews.
Proctor (1998) UK	To identify and compare the perceptions of women and midwives concerning women’s beliefs about what constitutes quality in maternity services.	Midwives (*n* = 47) Women (*n* = 33) Two large maternity units	Qualitative design	Focus group interviews Thematic analysis	An understanding of the concerns of women by maternity care staff is important in the development of a woman-centred service.
Rominov et al., (2017) Australia	To describe midwives, perceptions and experiences of engaging fathers in perinatal services.	Midwives (*n* = 106) Practising midwives (public and private)	Multi-method approach	Semi-structured interviews (*n* = 13) Descriptive analysis andsemantic thematic analysis	Midwives unanimously agreed that engaging fathers is part of their role and acknowledged the importance of receiving education to develop knowledge and skills with regard to fathers.
Sword et al., (2012) Canada	To explore women’s and care providers’ perspectives of quality prenatal care to inform the development of items for a new instrument.	Prenatal care providers (*n* = 40) Pregnant women (*n* = 40) Five urban centres across Canada	Qualitative descriptive approach	Semi-structured interviews Thematic analysis	A recurrent theme woven throughout the data reflected the importance of a meaningful relationship between a woman and her prenatal care provider that was characterized by trust.
Whitford et al., (2014) UK	To consider the use of a standard birth plan section within a national, woman-held maternity record.	Women (*n* = 42) Midwives (*n* = 15) Obstetricians (*n* = 6) GPs (*n* = 3) Antenatal clinics	Exploratory qualitative and longitudinal study	Interviews Thematic analysis	Staff and women were generally positive about the provision of a birth plan. Staff recognized the need to support women in completing their birth plan, but noted practical challenges concerning this.
Wright et al., (2018) Australia	To reveal how midwives enact woman-centred care in practice.	Midwives (*n* = 16) Public antenatal clinics and antenatal consultations	Contempo rary focused ethnography	Interviews and observation Thematic analysis	The ways in which midwives interacted with women during routine antenatal care demonstrated that some practices in a hospital setting can either support or undermine a woman-centred philosophy.

## Appendix A

**Table A1:** The eMERGe meta-ethnography reporting guidance.

Criteria Headings	Reporting Criteria	Pages
**Phase 1 Selecting meta-ethnography and getting started** *Introduction* 1. Rationale and context for the meta-ethnography 2. Aim(s) of the meta-ethnography 3. Focus of the meta-ethnography 4. Rationale for using meta-ethnography	Describe the gap in research or knowledge to be filled by the meta-ethnography and the wider context of the meta-ethnography Describe the meta-ethnography aim(s) Describe the meta-ethnography review question(s) (or objectives) Explain why meta-ethnography was considered the most appropriate qualitative synthesis methodology	2–3
**Phase 2 Deciding what is relevant** *Methods* 5. Search strategy 6. Search processes 7. Selecting primary studies 8. Outcome of study selection	Describe the rationale for the literature search strategy Describe how the literature searching was carried out and by whom Describe the process of study screening and selection, and who was involved Describe the results of study searches and screening	4–5
**Phase 3 Reading included studies** *Methods* 9. Reading and data-extraction approach *Findings* 10. Presenting characteristics of included studies	Describe the reading and data-extraction method and processes Describe characteristics of the included studies	5–7 7–11
**Phase 4 Determining how studies are related** *Methods* 11. Process for determining how studies are related *Findings* 12. Outcome of relating studies	Describe the methods and processes for determining how the included studies are related: -Which aspects of studies were compared -How the studies were compared Describe how studies relate to each other	11
**Phase 5 Translating studies into one another** *Methods* 13. Process of translating studies *Findings* 14. Outcome of translation	Describe the methods of translation: -Describe steps taken to preserve the context and meaning of the relationships between concepts within and across studies -Describe how the reciprocal and refutational translations were conducted -Describe how potential alternative interpretations or explanations were considered in the translations -Describe the interpretive findings of the translation	7,11 11–16
**Phase 6 Synthesizing translations** *Methods* 15. Synthesis process *Findings* 16. Outcome of synthesis process	Describe the methods used to develop overarching concepts (‘synthesized translations’), and describe how potential alternative interpretations or explanations were considered in the synthesis Describe the new theory, conceptual framework, model, configuration, or interpretation of data developed from the synthesis	11 11–12
**Phase 7 Expressing the synthesis** *Discussion* 17. Summary of findings 18. Strengths, limitations and reflexivity 19. Recommendations and conclusions	Summarize the main interpretive findings of the translation and synthesis, and compare them to existing literature Reflect on and describe the strengths and limitations of the synthesis: Methodological aspects: for example, describe how the synthesis findings were influenced by the nature of the included studies and how the meta-ethnography was conducted Reflexivity—for example, the impact of the research team on the synthesis findings Describe the implications of the synthesis	11–16 18

**Table A2:** CERQual Qualitative Evidence Profile. Check row alignment in this table

Review Finding		Studies Contributing to the Review Finding	Assessment of Methodological Limitations	Assessment of Relevance	Assessment of Coherence	Assessment of Adequacy	Overall CERQual Assessment of Confidence	Explanation of Judgement
Will to change from routine ANC to family-centred ANC	Lack of support from colleagues Lack of cultural understanding Lack of confidence, knowledge and skills to support new practices Lack of willingness to learn and try group-based care	Andersson Ahlden Ahlden Andersson Aquino Goodwin Rominov Ahlden Andersson Dalton Sword Andersson	Minor methodological limitations. All studies but two lacked clarity about reflexivity. Two studies had minor concerns about data analysis.	Minor concerns about relevance. Nurse-midwives/midwives working at antenatal clinics in community/hospital setting in four countries.	Minor concerns regarding coherence. Data reasonably consistent within and across studies.	Minor concerns about adequacy of data. Six studies presented moderate or rich data.	Moderate confidence.	The finding was graded as moderate confidence because of minor methodological considerations, minor concerns about relevance, coherence and adequacy of data.
Need to change rigid systems that maintain routine care	Frustrated by unfit premises, restricted access to ICT and interpret ting service Frustrated by rigid structures, risk aversion and medicalization Frustrated by time pressure and cultural misunderstandings	Andersson Aquino Dalton Proctor Rominov Sword Ahlden Dove McCourt Wright Ahlden Aquino Browne Goodwin McCourt Whitford Wright	Minor methodological limitations. Two studies had minor concerns about data analysis. All studies but two lacked clarity about reflexivity. One study lacked clarity about ethical considerations.	Minor concerns about relevance.	Minor concerns regarding coherence. Data reasonably consistent within and across studies.	Moderate concerns about adequacy of data. Six studies presented rich data. Four studies presented moderate data, two studies presented thin data.	Moderate confidence.	The findings were graded as moderate confidence because of minor methodological limitations, minor concerns about relevance and coherence and moderate concern about data adequacy in two studies.
Readiness for midwife-led- and family-centred models of ANC	Readiness to use ICT to complement personal encounters Readiness to listen to parents’ questions and requests for group-based care Readiness for individual continuity of care Readiness to include fathers and family members	Dalton Rominov Andersson Ahlden Aquino Olsson Andersson Browne Dove McCourt Olsson Sword Wright Ahlden Browne Goodwin Proctor Rominov Sword	Minor methodological limitations. Two studies had minor concerns about data analysis. All studies but two lacked clarity about reflexivity. One study lacked clarity about ethical considerations.	Minor concerns about relevance.	Minor concerns regarding coherence. Data reasonably consistent within and across studies.	Minor concerns about adequacy of data.	Moderate confidence.	The findings were graded as moderate confidence because of minor methodological limitations and minor concerns about relevance and coherence.

Definitions of levels of confidence from the CERQual evaluation: High confidence: It is highly likely that the review finding is a reasonable representation of the phenomenon of interest. Moderate confidence: It is likely that the review finding is a reasonable representation of the phenomenon of interest. Low confidence: It is possible that the review finding is a reasonable representation of the phenomenon of interest. Very low confidence: It is not clear whether the review finding is a reasonable representation of the phenomenon of interest.
